# Remarks on Muscle Contraction Mechanism II. Isometric Tension Transient and Isotonic Velocity Transient

**DOI:** 10.3390/ijms12031697

**Published:** 2011-03-04

**Authors:** Toshio Mitsui, Nobukatsu Takai, Hiroyuki Ohshima

**Affiliations:** 1 Osaka University, 1-1 Yamadaoka, Suita, Osaka, 565-0871, Japan; 2 Faculty of Engineering, Hokkai-Gakuen University, S26 W11, Chuo-ku, Sapporo, 064-0926, Japan; E-Mail: takai@eli.hokkai-s-u.ac.jp; 3 Faculty of Pharmaceutical Sciences, Tokyo University of Science, 2641, Yamazaki, Noda, Chiba, 278-8510, Japan; E-Mail: ohshima@rs.noda.tus.ac.jp

**Keywords:** muscle contraction mechanism, theory, isometric tension transient, isotonic velocity transient, double-hyperbolic force-velocity relation, crossbridge extensibility, filament extensibility, actomyosin complex, U* transition, U*_12_ transition

## Abstract

Mitsui and Ohshima (2008) criticized the power-stroke model for muscle contraction and proposed a new model. In the new model, about 41% of the myosin heads are bound to actin filaments, and each bound head forms a complex MA_3_ with three actin molecules A1, A2 and A3 forming the crossbridge. The complex translates along the actin filament cooperating with each other. The new model well explained the experimental data on the steady filament sliding. As an extension of the study, the isometric tension transient and isotonic velocity transient are investigated. Statistical ensemble of crossbridges is introduced, and variation of the binding probability of myosin head to A1 is considered. When the binding probability to A1 is zero, the Hill-type force-velocity relation is resulted in. When the binding probability to A1 becomes finite, the deviation from the Hill-type force-velocity relation takes place, as observed by Edman (1988). The characteristics of the isometric tension transient observed by Ford, Huxley and Simmons (1977) and of the isotonic velocity transient observed by Civan and Podolsky (1966) are theoretically reproduced. Ratios of the extensibility are estimated as 0.22 for the crossbridge, 0.26 for the myosin filament and 0.52 for the actin filament, in consistency with the values determined by X-ray diffraction by Wakabayashi *et al.* (1994).

## 1. Introduction

In 1999, Mitsui [1] criticized the power-stroke model on the muscle contraction mechanism and proposed a new model. In 2008, Mitsui and Ohshima [2] refined the new model and discussed the steady filament sliding in detail demonstrating that the calculation results were in good agreement with experimental observations. They also outlined the discussion on the isometric tension transient and the isotonic velocity transient given in [1] by citing some calculation results. Thereafter, however, a few readers of [1] commented that it was very difficult to understand the discussion on the transient phenomena in [1], since there was no detailed explanation on the molecular processes upon which the theoretical treatment was based. In the present paper, we have largely revised that part of [1], trying to make the discussion more readable.

Now the article [2] is regarded as Part I of Remarks series, in which the basic ideas of our model are introduced and steady muscle behaviors are discussed. In the present paper (Remarks II), non-steady muscle behaviors are discussed. We are preparing an article as Remarks III, in which discussion will be done on more recent experimental studies as cited in the articles [3,4].

The basic ideas of our model introduced in [2] are summarized as follows. A simple thermodynamic relation is derived, which indicates that there is an inconsistency in the power stroke model or swinging lever model. Our model is proposed to avoid this difficulty. It is assumed that a myosin head forms a complex with three actin molecules when it attaches to an actin filament. Here it should be noted that Andreeva *et al.* [5] found the evidence that the crossbridge can interact with more than 1 actin monomer. The complex corresponds to the crossbridge. According to the X-ray diffraction studies [6–9], the intensity ratio of the [1, 0] and [1, 1] equatorial reflections increases only minimally as the shortening velocity increases, indicating that the total number of myosin heads in the vicinity of the actin filament decreases only slightly. Taking this fact into account, it is assumed that about 41% of the myosin heads forms the crossbridges at any sliding velocity. Then mutual cooperativity takes place among the crossbridges in filament sliding, so that energy dissipation becomes reasonable magnitude (the order of *kT*) for one step of the crossbridge movement. Calculation based upon the model well reproduce the force-velocity relation given by Hill [10] and the energy liberation rate *vs.* force relation given by Hill [11].

Since the present study is based upon ideas that are quite different from the power stroke model and others, the basic ideas of our study are explained in some detail in Section 2. In Section 3, discussion is done of how variation in the crossbridge binding affects the muscle tension. In Section 4, discussion is done on the difference between the molecular processes in Phases 1 of the isometric tension transient and of the isotonic velocity transient, and extensibility ratios for the crossbridge, the myosin filament and the actin filament are estimated. Time course of the isometric tension transient is studied in Section 5. Time course of the isotonic velocity transient is studied in Section 6. The deviation from the Hill-type force-velocity relation is derived in Section 7. Obtained results are summarized and discussed in Section 8.

## 2. Basic Ideas for Discussion of the Transient Phenomena

### 2.1. Deviation from the Hill-Type Force-Velocity Relation

**Figure 1.**
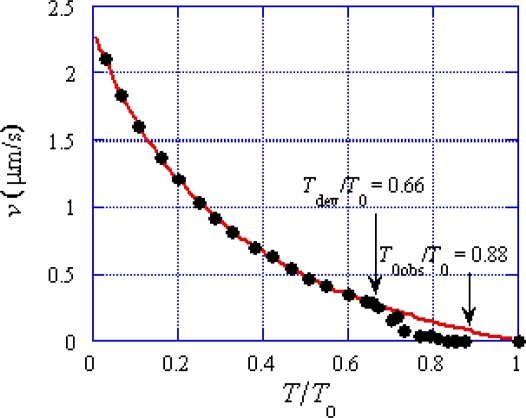
Force-velocity relation and definition of *T*_dev_ and *T*_0obs_. The red curve is the calculation result reported in [2], which coincides with the hyperbolic force-velocity relation proposed by Hill [10]. Circles are the experimental results cited from Figure 6A of the paper by Edman [12].

In the present study, the deviation from the hyperbolic force-velocity relation shown in Figure 1 has very important implication. The red line is our calculation results reported in [2] which agrees with the empirical hyperbolic force-velocity relation proposed by Hill [10]. The curve will be called Hill-type below. Edman [12], however, reported that carefully measured velocity deviated from the Hill-type relation as given by the black circles in Figure 1, which he called a double-hyperbolic force-velocity relation. In Figure 1, the tension *T* at which the experimental data start to deviate from the Hill-type curve is indicated as *T*_dev_ and the tension *T* at which *T* becomes 0 is denoted as *T*_0obs_. The following values are determined by the data presented in Figure 6A of [12].

(1) $$T_{\text{dev}}/T_{0}=0.66$$

(2) $$T_{0\text{obs}}/T_{0}=0.88$$

### 2.2. MA_3_ Complex and U*_12_ Transition

**Figure 2.**
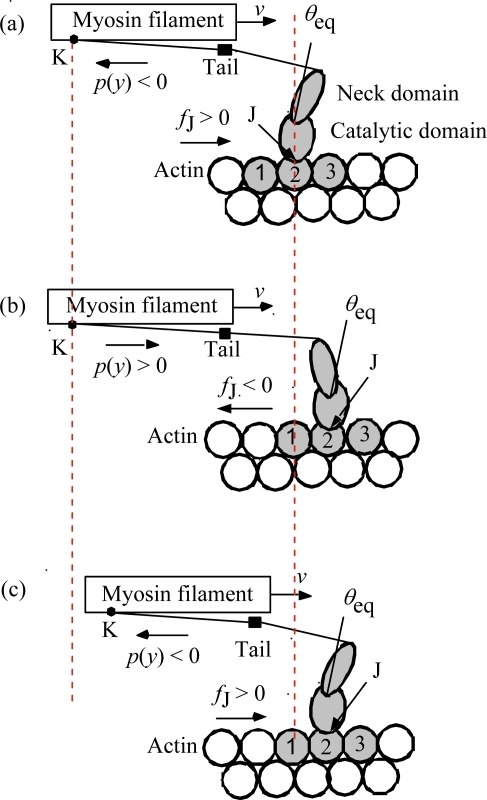
MA_3_ complex (shadowed) and its step motion along the actin filament (after Figure 6 of [2]). A myosin head is expressed as neck domain plus catalytic domain and the actin molecules in MA_3_ as 1, 2 and 3. **(a)** Just before the myosin head moves to the right; **(b)** Just after the head (and the complex MA_3_) moves to the new position; **(c)** The head is ready for next movement to the right. (Note that the actin molecule bound to a myosin molecule is called 2.)

In our model, about 41% of the myosin heads are bound to actin filaments (cf. Equation 3–1–1 in [2]), and each bound head forms a complex MA_3_ with three actin molecules. The complex MA_3_ translates along the actin filament changing the partner actin molecules. Figure 2 illustrates the step motion of MA_3_. The translation of the crossbridge along the actin filament is made possible by the structural change of MA_3_ induced by the force *f*_J_ which exerts on the junction J between the crossbridge and the actin filament.

**Figure 3.**
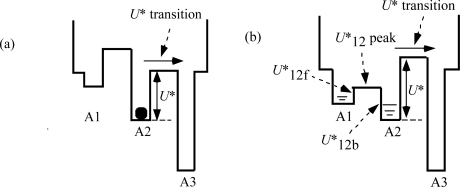
Potential of the force exerted on the myosin head in the MA_3_ complex. The shape varies depending upon the tension *T*. **(a)** The case of *T* < *T*_dev_, where the force-velocity relation is Hill-type. The black circle indicates that the myosin head exists solely in well A2; **(b)** The case of *T*_dev_ < *T* < *T*_0obs_, where the existence probability of the myosin head is distributed in wells A1 and A2, causing deviation of the force-velocity relation from Hill-type.

Figure 3 shows the distribution of potential of force exerted on the myosin head in MA_3_. There are three potential wells on the actin filament corresponding to the three binding sites A1, A2 and A3. The wells are also as called A1, A2 and A3. Figure 3(a) shows the potential distribution considered in [2], where a myosin head exists solely in well A2. It is assumed that the potential barrier U* depends upon the force *f*_J_ by U* = U*_0_ − *af*_J_ (Equation 3–5–2 in [2])). The kinetics of the myosin head will be discussed based upon Eyring’s theory of the rate process (cf. [13]) as was done by Huxley and Simmons [14] and in [2]. Then the probability that the myosin head moves from well 2 to 3 across the potential barrier U* becomes proportional to exp(−U*/*kT*). Calculation was done in [2] based upon the model in Figure 3(a). An example of calculation results is cited by the red curve in Figure 1. Since the calculation well explains the experimental data for *T* < *T*_dev_. this model seems close to reality when *T* < *T*_dev_. The deviation from the Hill-type relation when *T* > *T*_dev_ suggests that the model in Figure 3(a) should be modified, and the model in Figure 3(b) becomes the object to be considered in the present study. It is assumed that myosin heads start to move over the potential barrier U*_12_ when *T* becomes larger than *T*_dev_, and heads are distributed in A1 and A2 when *T* > *T*_dev_. The potential peak between A1 and A2 will be called U*_12_ and the potential barrier for backward movement from A2 to A1 will be denoted as U*_12b_ and the barrier for forward movement from A1 to A2 as U*_12f_. In the followings, transitions of these types will be called U*_12_ transition.

The displacement of the myosin head from A2 to A1 was briefly mentioned in Section 3.6 of [2] in term of “pull-up transition”. In the article [1], the isometric tension transient was discussed in relation with the U*_12_ transition by the statistical mechanics. Although the present study is based upon the same idea, we have found that such statistical-mechanical approach as in [1] makes the discussion very complex. We shall discuss the problem in a different manner as described below.

### 2.3. Definition of Crossbridge Shortening Y

**Figure 4.**
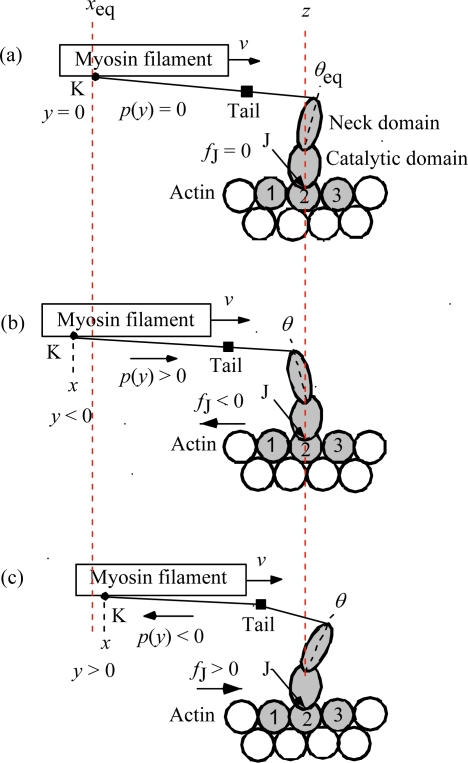
Definition of *y*, the shortening of the crossbridge: *y* = *x* − *x*_eq_ (after Figure 4 of [2]). The binding positions J are set on the dotted red line *z*. **(a)** The myosin head is at its equilibrium angle, *θ*_eq_. *x* = *x*_eq_ and *y* = 0; **(b)** The myosin head is pulling the myosin filament forward. The crossbridge is elongated and *y* < 0; **(c)** The myosin head is pushing the myosin filament backward. The crossbridge is shortened and *y* > 0.

In the following discussion, a parameter *y* is frequently used to represent shortening (which is positive for negative length change) of the crossbridge as in [2]. Unfortunately, the symbol *y* was used to represent elongation of the crossbridge in [1]. In the present paper, as an extension of the discussion of [2], *y* represents shortening. Figure 4 is to make the definition of *y* clear. K and J, respectively, are ends of the crossbridge on the myosin and actin filaments. The symbol *x* is defined as the projection of the vector from K to J on the myosin filament. Since the positions J are set on the right-handed red dotted line indicated as z in Figure 4, *x* is determined by the position of K. As shown in Figure 4(a), *x* is denoted as *x*_eq_ when the tilting angle of the myosin head neck domain is at the equilibrium angle *θ*_Equation_. Then *y* is defined by

(3) $$y=x-x_{\text{eq}}$$

The red dotted line indicated as *x*_eq_ in Figure 4 indicates the origin of *y*. Thus *y* = 0 in Figure 4(a), *y* < 0 in (b) and *y* > 0 in (c). As discussed in [2], the crossbridge with shortening *y* exerts the force *p*(*y*) on the myosin filament. The stiffnesses for forward and backward forces are denoted as *κ*_f_ and *κ*_b_, and, according to Equation 3–4–6 in [2], *p*(*y*) is given by

(4a) $$p(y)=-\kappa_{\text{f}}y\;\text{for}\;y<0$$

(4b) $$p(y)=-\kappa_{\text{b}}y\;\text{for}\;y>0$$

Values of *κ*_f_ and *κ*_b_ are given in Appendix.

### 2.4. Time Constant of the U* Transition

**Figure 5.**
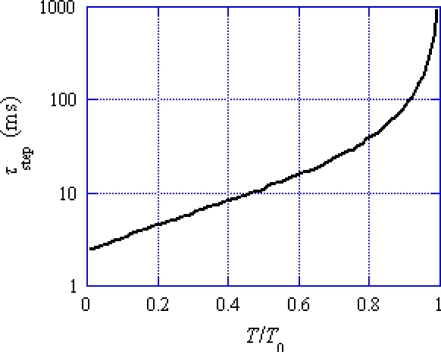
Time interval *τ*_step_ for a step motion of the myosin head as a function of *T*/*T*_0_. *τ*_step_ = *L*/*v*, which is approximately equal to the time interval of the occurrence of the U* transition.

To discuss the transition phenomena, it is important to have an idea about the mean time interval of occurrence of the U* transition. In the steady filament sliding, the mean time interval is equal to the mean time *τ*_step_ in which a myosin head moves from one actin molecule to the neighboring one. This *τ*_step_ is given by

(5) $$\tau_{\text{step}}=L/v$$

where *L* is the distance between the centers of actin molecules 2 and 3 in Figure 2 (the value of *L* is given in Appendix) and *v* is the sliding velocity. Values of *τ*_step_ are calculated by using *v* given by the red curve in Figure 1, and shown as a function of the relative tension in Figure 5, which indicates that *τ*_step_ is about 2 ms at *T*/*T*_0_ = 0, becomes 10 ms around *T*/*T*_0_ = 0.5 and increases to 1 s around *T*/*T*_0_ = 1. This result means that U* transition generally does not contribute to the early processes in the transition phenomena.

### 2.5. Statistical Ensemble of Crossbridges and Crossbridge Binding Probability ρ

In discussion of the steady filament sliding [2], the crossbridge shortening *y* repeats the cycle from *y*_c_-*L* to *y*_c_ through filament sliding and from *y*_c_ to *y*_c_-*L* through the U* transition (cf, Figure 7 in [2]), where *y*_c_ is the parameter which decreases as the tension *T* increases (as shown in Figure 8 given later.) In the following discussion, this temporal scheme is replaced by a spatial scheme. In our model, the ratio *r* = (number of myosin heads bound to actin filaments)/(total number of myosin heads) is constant independent of tension (*r* = 0.41, cf. Appendix). Accordingly, we can consider an ensemble of bound crossbridges of definite number independent of the tension.

Suppose that all complexes MA_3_ in right half sarcomeres are collected and superposed putting A2 at the same position. This ensemble can be characterized by the ratio *ρ*(*y*)d*y* = (number of crossbridges having shrinkage between *y* and *y* + d*y*)/(total number of crossbridges). By definition, integration of *ρ*(*y*) is 1. Actually *ρ*(*y*) is a smooth function. The mean value of *y* in the *ρ* distribution is denoted as <*y*> and *y*_c_ is defined by

(6) $$y_{\text{c}}=<y>+(L/2)$$

Then *ρ*(*y*) is expected to be large between *y*_c_-*L* and *y*_c_. In the following discussion, *ρ*(*y*) is approximated to be constant (1/*L*) between *y*_c_-*L* and *y*_c_ and zero outside of the region. This will be called rectangular *ρ* approximation. Equivalent approximation was used in the temporal scheme in [2] for studies of the steady filament sliding.

The force-velocity relation deviates from Hill-type for *T* > *T*_dev_ as shown in Figure 1. The origin of the deviation is considered as follows. In Figure 3, the potential of force exerted on the myosin head in the MA_3_ complex is shown. The potential at A2 is lower than that at A1, and all the myosin heads are present in well A2 in the case of *T* < *T*_dev_ as shown in Figure 3(a). On the other hand, as seen in Figure 4(b), the backward force exerted on the myosin head becomes stronger as *y* negatively increases, so that the myosin heads having negatively large *y* tend to bind to A1 through the U*_12_ transition. Accordingly, the distribution of the myosin heads becomes as symbolically shown in Figure 3(b) for *T* > *T*_dev_. This effect is regarded as the origin of the deviation of force-velocity relation from the Hill-type.

Symbols *y*_0obs_ and *y*_cdev_ are defined as *y*_c_’s at *T*_0obs_ and *T*_dev_, respectively, on the Hill-type force-velocity relation (cf. Figure 8 given later.) As mentioned above, *y*_c_ decreases with increasing *T*, and thus *T* < *T*_dev_ corresponds to *y*_c_ > *y*_cdev_.

**Figure 6.**
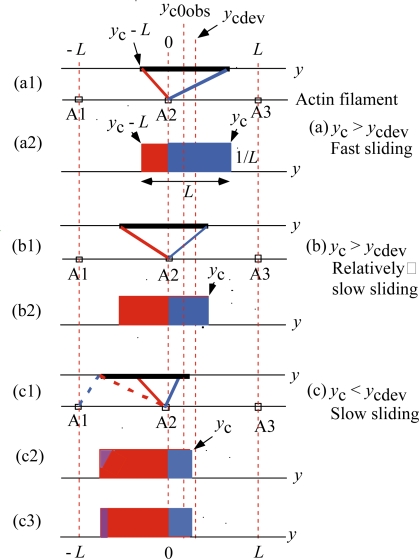
Statistical ensemble of crossbridges in the steady filament sliding.

Figure 6 shows examples of the rectangular *ρ* distribution for the steady filament sliding. Figure 6(a) is for fast sliding and (b) is for relatively slow sliding in the case of *y*_c_ > *y*_cdev_ or *T* < *T*_dev_. Figure 6(c) is for slow sliding in the case of *y*_c_ < *y*_cdev_ or *T* > *T*_dev_. In Figures 6(a1), (b1) and (c1), the upper horizontal line is an axis of the crossbridge shortening *y* and the black thick segment on it shows the regions of finite *ρ*(*y*) of length *L*. The lower horizontal lines correspond to the actin filament and A1, A2 and A3 represent actin molecules. The crossbridges having negative *y* produce positive stress *p*(*y*) according to Equation 4a. The red segments with their ends at A2 are examples of such crossbridges. The crossbridges having positive *y* produce negative stress *p*(*y*) according to Equation 4b. The blue segments with their ends at A2 or A1 are examples of such crossbridges.

Figures 6(a2) and (b2) show the *ρ* distributions as functions of *y*, *ρ*(*y*) by the rectangular approximation, where *ρ*(*y*) is equal to (1/*L*) between *y*_c_-*L* and *y*_c_, and equal to zero outside of the region. Crossbridges in the red area produce positive stress, and crossbridges in the blue area produce negative stress. As the edge *y*_c_ shifts to the left from (a2) to (b2), the red region increases and the crossbridge ensemble produce more stress and the filament sliding becomes slow (Note *κ*_f_ > *κ*_b_ in Appendix).

Figure 6(c) corresponds to the case of *T* > *T*_dev_ and thus *y*_c_ < *y*_cdev_. In (c1), the blue dotted segment symbolically indicates that a portion of crossbridges of small *y* bind to A1 and produces negative tension, while the red dotted segment indicates that the rest of crossbridges having the same small *y* still bind to A2 and produce positive tension. In (c2), the violet triangle symbolically indicates that the crossbridges in this triangle are bound to A1. As shown in (c3), the violet triangle is replaced by the violet rectangle containing the same number of the crossbridges, to make calculation easy. In the following calculation, it is assumed that all the crossbridges in the violet rectangle are bound to A1 and produce negative tension.

Now let us calculate values of *y*_cdev_ and *y*_c0obs_. By definition, *y*_cdev_ and *y*_c0obs_ are *y*_c_’s for *T*_0obs_ and *T*_dev_ in the Hill-type force-velocity relation. The mean tension per one crossbridge is denoted as *p* in [2], which can be obtained by integration of the tension *p*(*y*). Calculation is done by using *p*(*y*) = −*κ*_f_*y*/*L* (Equation 4a) in the red region and *p*(*y*) = −*κ*_b_*y*/*L* (Equation 4b) in the blue region in Figure 6 (a2) or (b2). Result of the integration is

(7) $$p=\{-(-\kappa_{\text{f}}/2){\left(y_{\text{c}}-L\right)}^{2}+(-\kappa_{\text{b}}/2)y_{\text{c}}^{2}\}/L$$

This relation leads us to the Hill-type force-velocity relation in cooperation with the U* transition [2]. The isometric tension in the Hill-type relation is denoted as *p*_0_ and *T*_0_ following [2]. Then, by using *p*/*p*_0_ = *T*/*T*_0_ (Equation 3–1–2 in [2]), the relative tension *T*/*T*_0_ is given by

(8) $$T/T_{0}=\{-(-\kappa_{\text{f}}/2){\left(y_{\text{c}}-L\right)}^{2}+(-\kappa_{\text{b}}/2)y_{\text{c}}^{2}\}/({\text{Lp}}_{0})$$

This equation gives relations between *T*_dev_ and *y*_cdev_ and between *T*_0obs_ and *y*_c0obs_. Combining Equations 1, 2 and 8 gives

(9) $$y_{\text{cdev}}=1.60\;\text{nm},$$

(10) $$y_{\text{c}0\text{obs}}=1.03\;\text{nm}.$$

In Figure 6, *y*_cdev_ and *y*_c0obs_ are indicated by the vertical dotted red lines.

## 3. Effect of U*_12_ Transition on the Tension

Equation 8 leads us to the Hill-type force-velocity relation. The stress *T* and *y*_c_ related by Equation 8 is denoted as *T*^H^ and *y*_c_^H^, which are in agreement with the observation when *y*_c_^H^ > *y*_cdev_. The observed *T* and *y*_c_ are denoted as T* and *y*_c_* when *y*_c_^H^ < *y*_cdev_.

**Figure 7.**
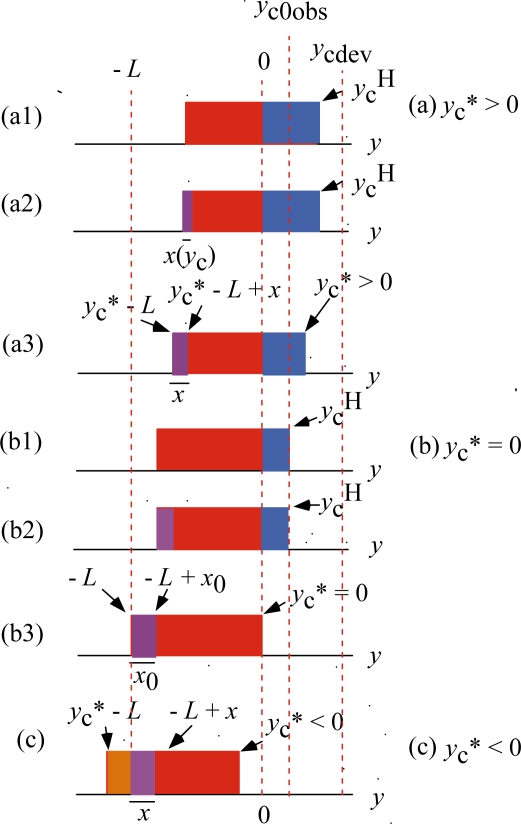
Compensation of the effect of the U*_12_ transition by shift of *y*_c_^H^ to *y*_c_*. Magnitudes of *y*_c0obs_ and *y*_cdev_ are exaggerated for illustration. **(a)** *y*_c0obs_ < *y*_c_^H^ < *y*_cdev_ and *y*_c_* > 0; **(b)** *y*_c_* = 0; **(c)** *y*_c_* < 0.

Figure 7 illustrates the *ρ* distributions in various cases of *y*_c_^H^ < *y*_cdev_. Figure 7(a1) shows the *ρ* distribution by Equation 8 having the edge *y*_c_^H^. This *ρ* distribution gives *T*^H^ even though *y*_c_ < *y*_cdev_. It is an imaginary state in which the U*_12_ transition is absent. Actually, however, the U*_12_ transition takes place and the violet area appears causing decrease of the red area as shown in (a2), where the width of the violet area is denoted as *x*(*y*_c_). Naturally *T* in (a2) is smaller than *T* in (a1). Then *y*_c_ in (a2) is changed into *y*_c_* in (a3) to make *T* the same as *T* in (a1), where the width of the violet region is denoted as *x*. The change of the blue area from *y*_c_ to *y*_c_* causes an increase of the tension while the change of the violet area from *x*(*y*_c_) to *x* causes an decrease of the tension. If the effect of the former change is larger than that of the latter change, the tension in (a2) will increase and can be the same as in (a1). Below we shall assume that such tension adjustment actually takes place and discuss the relation between *y*_c_* and T*.

Since the tension is the same in Figures 7(a1) and (a3), the blue area in (a1) is the same as the sum of the blue area and violet area in (a3). Figures 7(b1)–(b3) show the case of y* = 0. Similarly to (a1) and (a3), the blue area in (b1) is the same as the violet area in (b3). The width *x* of the violet area in (b3) is denoted as *x*_0_. Figure 7 (c) shows the case that *y*_c_* becomes negative. The brown area means that crossbridges in this area bind to A1 and produce positive stress. The width of the violet area is indicated as *x*. Extrapolating the change from *x* in (a3) to *x*_0_ in (b3), *x* in (c) is assumed to be larger than *x*_0_. Then sum of the red area and brown area in (c) is smaller than red area in (b3) and thus the tension produced in (c) is smaller than the tension in (b3). Then the stress produced in (b3) is the maximum that the muscle machine can produce, and should be equal to *T*_0obs_. Accordingly, *y*_c_^H^ in (b1) should be equal to *y*_c0obs_. Since the red area in (b1) is the same as the red area in (b3), *y*_c_^H^ in (b1) is equal to *x*_0_ in (b3). Thus we have

(11) $$x_{0}=y_{\text{c}0\text{obs}}$$

The tension T* deviates from *T*^H^ at *y*_c_ = *y*_c_* = *y*_cdev_, and reaches its maximum at *y*_c_* = 0. To express such characteristics of T* vs. y* relation, the following set of equations are used:

(12) $$T^{*}/T_{0}=-{\text{ay}}_{c}^{{*}^{2}}+b$$

(13) $$a=(T_{0\text{obs}}-T_{\text{dev}})/(T_{0}y_{\text{cdev}}^{2})=0.086\;{\left(\text{nm}\right)}^{-2}$$

(14) $$b=T_{0\text{obs}}/T_{0}=0.88$$

**Figure 8.**
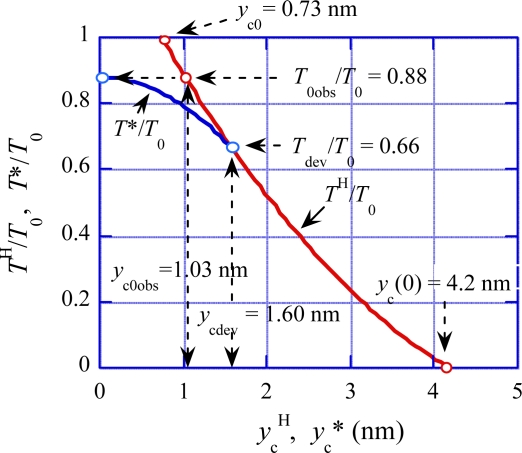
Relative tensions as functions of *y*_c_. Red line: *T*^H^/*T*_0_ as a function of *y*_c_^H^. Blue line: T*/*T*_0_ as a function of *y*_c_*. (For definition of *y*_c0_ and *y*_c_(0), see Appendix).

In Figure 8, the blue curve illustrates the T*/*T*_0_ *vs. y*_c_* relation given by this set of equations, and the red curve shows the Hill-type *T*^H^/*T*_0_ *vs. y*_c_^H^ relation. The blue curve deviates from the red curve at *y*_c_ = *y*_c_* = *y*_cdev_ and exhibits its maximum at *y*_c_* = 0 where T^*^/*T*_0_ = *T*_0obs_/*T*_0_.

Up to here, *T*/*T*_0_ is used for the relative tension, where *T*_0_ is the maximum tension in the extended Hill-type force-velocity relation. The experimentally observed maximum tension, however, is *T*_0obs_ where *T*_0obs_ = 0.88*T*_0_ (Equation 2). Hereafter discussion is concerned with experimental data and relative tension is defined as *T*/*T*_0obs_. In our model *T*/*T*_0_ = *p*/*p*_0_ (Equation 3–1–2 in [2]), and the force *p* corresponding to *T*_0obs_ is denoted as *p*_0obs_. Then

(15) $$p_{0\text{obs}}=0.88\;p_{0}$$

and

(16) $$T/T_{0\text{obs}}=p/p_{0\text{obs}}$$

If we put *y*_c_ = *y*_c0obs_ in Equation 7 and use *x*_0_ = *y*_c0obs_ (Equation 11), we get theoretical expression of *p*_0obs_ as

(17) $$p_{0\text{obs}}=\{-(-\kappa_{\text{f}}/2){\left(-L+x_{0}\right)}^{2}+(-\kappa_{\text{b}}/2)x_{0}^{2}\}\;/L$$

## 4. Phases 1 in the Isometric Tension Transient and Isotonic Velocity Transient, and Extensibility Ratios for the Crossbridge, Myosin Filament and Actin Filament

Huxley [15] divided the transient responses to the sudden reduction of length or of load into four Phases. In our model, however, there is no exact correspondence between molecular processes of the four Phases in the tension transient and those in the velocity transient. To avoid confusion, we use the terms, Phase Tn in the tension transient and Phase Vn in the velocity transient, where n = 1, 2, 3, 4.

**Figure 9.**
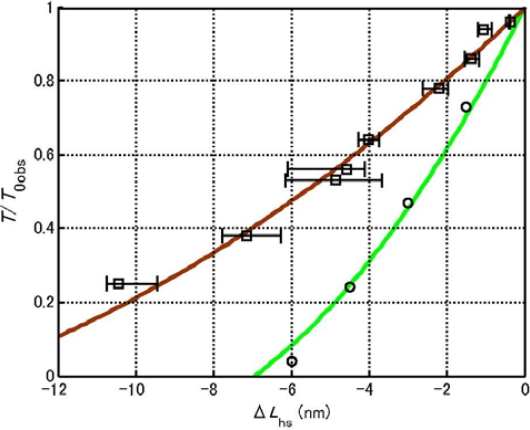
*T*/*T*_0obs_ *vs.* Δ*L*_hs_ relations in Phase T1 and Phase V1. Circles: experimental data cited from Figure 13 of Ford *et al.* [16]. Squares: experimental data obtained from Figure 3 of Civan and Podolsky [17], in which the error bar means error of read-out from Figure 3 of [17]. The green curve: *T*/*T*_0obs_ calculated by Equations 18 and 20 with *C*_CBT_ = 2.2 (Equation 22). The brown curve: *T*/*T*_0obs_ calculated by Equations 19 and 21, with *C*_CBV_ = 4.6 (Equation 23).

Phase T1 is simultaneous decrease of tension in the isometric tension transient, while Phase V1 is simultaneous shortening of muscle in the velocity transient. The length change per half sarcomere is denoted as Δ*L*_hs_ in both transients. The experimentally measured isometric tension is *T*_0obs_ and the relative tension is defined by *T*/*T*_0obs_. The relative load used in the paper by Civan and Podolsky [8] is expressed as the relative tension *T*/*T*_0obs_ below. Figure 9 shows the experimental results on Phases T1 and V1, by circles for the isometric tension transient cited from the paper by Ford *et al.* [16] and by squares for the isotonic velocity transient cited from the paper by Civan and Podolsky [17].

At first sight, it was puzzling to see that distributions of the experimental data are quite different for the two cases, since the structural changes seem to be purely elastic both in Phases T1 and V1. Then it was reminded that the length change of sarcomere is a sum of those of the crossbridge, myosin filament and actin filament, which are proportional to each other (cf. the review by Irving [18]). This means that there are three elastic components, and it is a possibility that they have different response times in elastic changes from each other.

In this connection, the experimental data reported by Julian and Sollins [19] seem important. They measured tension changes of single frog skeletal muscle fiber at increasing speed of step shortening. Their Figures 2 and 4 show experimental data on relative force *vs.* Δ*L*_hs_ relation at different speeds of shortening. The distribution of open circles in their Figure 2 (the length change period of about 1 ms) is similar to that of the data by Civan and Podolsky (1∼2 ms) cited in our Figure 9. The distribution of filled triangles in Figure 4 of [9] (length change period of 0.4 ms) is close to the data by Ford *et al.* (the length change period of 0.2 ms) cited in our Figure 9. These facts seem to indicate that there are two kinds of elastic process in the tension response: The fast one occurs within about 0.4 ms and the slow one occurs between about 0.4 and 1 ms after the length change. The fast process seems responsible to the change in Phase T1 and combination of the fast and slow processes seems responsible to Phase V1.

**Figure 10.**
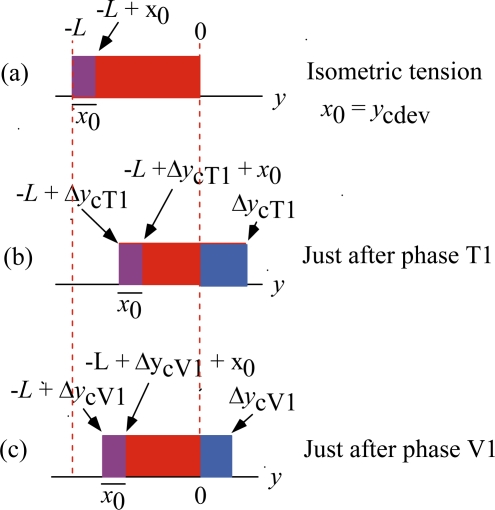
The *ρ* distributions before and after instantaneous changes in the isometric tension transient and in the isotonic velocity transient. **(a)** The isometric tetanus state; (**b**) Just after Phase T1 in the isometric tension transient; **(c)** Just after Phase V1 in the isotonic velocity transient for the same length change as **(b)**.

It seems plausible that elastic response of crossbridge and myosin filament almost simultaneously occurs since they belong to the same molecule and elastic response of actin filament occurs with some delay. Thus it is assumed that crossbridge and myosin filament are responsible to the change in Phase T1 and that all three components are responsible to the change in Phase V1. Now changes of *y*_c_ in Phases T1 and V1 should be different from each other even when the length change Δ*L*_hs_ is the same. Calculation is done on this assumption by using the rectangular *ρ* distribution shown in Figure 10.

Figure 10(a) shows the *ρ* distribution at the isometric tension (the same as Figure 7 (b3)). Figure 10(b) shows the *ρ* distribution just after Phase T1 and (c) the one just after Phase V1 for the same length change Δ*L*_hs_ as (b). The violet areas in Figure 10 (b) and (c) are the same as (a) since U*_12_ transition does not occur yet. Changes of the edge *y*_c_ in Phases T1 and V1 are denoted as Δ*y*_cT1_ and Δ*y*_cV1_. For the same Δ*L*_hs_, the tension *T* in Phase T1 is smaller than *T* in Phase V1 in Figure 9. The edge Δ*y*_cT1_ in Figure 10 (b) is set larger than Δ*y*_cV1_ in (c) so as to make the red area in (b) smaller than that in (c), in accordance with the fact that the tension *T* in Phase T1 is smaller than *T* in Phase V1.

Since elastic changes of the crossbridge, myosin filament and actin filament are proportional to each other [10], the Δ*y*_cT1_ in (b) and Δ*y*_cV1_ in (c) are proportional to Δ*L*_hs_, and expressed by

(18) $$\Delta y_{\text{cT}1}=-\Delta L_{\text{hs}}/C_{\text{CBT}}$$

and

(19) $$\Delta y_{\text{cV}1}=-\Delta L_{\text{hs}}/C_{\text{CBV}}$$

where *C*_CBT_ and *C*_CBV_ are constants.

Let the tensions in Figure 10(b) and (c) be denoted as *T*_T1_ and *T*_V1_, respectively. They are given by integration of −*κ*_b_{*y* − (−*L*)} in the violet area, −*κ*_f_*y* in the red area and −*κ*_b_*y* in the blue area. Thus we have

(20) $$T_{\text{T}1}/T_{0\text{obs}}=[(-\kappa_{\text{b}}/2)\{{\left(x_{0}+\Delta y_{\text{cT}1}\right)}^{2}-\Delta y_{\text{cT}1}^{2}\}-(-\kappa_{\text{f}}/2){\left(-L+x_{0}\Delta y_{\text{cT}1}\right)}^{2}+(-\kappa_{\text{b}}/2)\Delta y_{\text{cT}1}^{2}]/(Lp_{0\text{obs}})$$

and

(21) $$T_{\text{V}1}/T_{0\text{obs}}=[(-\kappa_{\text{b}}/2)\{{\left({\text{x}}_{0}+\Delta y_{\text{cV}1}\right)}^{2}-\Delta y_{\text{cV}1}^{2}\}-(-\kappa_{\text{f}}/2){\left(-L+x_{0}\;\Delta y_{\text{cV}1}\right)}^{2}+(-\kappa_{\text{b}}/2)\Delta y_{\text{cV}1}^{2}]/(Lp_{0\text{obs}})$$

Calculations were done for various trial values of *C*_CBT_ (Equation 18) and *C*_CBV_ (Equation 19). The best fit for the experimental data are obtained for the values

(22) $$C_{\text{CBT}}=2.2$$

(23) $$C_{\text{CBV}}=4.6$$

Figure 9 shows the calculation results for *T*_T1_/*T*_0obs_ by the green curve and for *T*_V1_/*T*_0obs_ by the brown curve. They are in good agreement with the experimental data.

Let us denote the ratios of extensibilities of the crossbridge, the myosin filament and the actin filament as *r*_CB_, *r*_M_, and *r*_A_ at the elastic equilibrium respectively. Shortenings of the three per half sarcomere are denoted, respectively, as Δ*y*_CB_, Δ*y*_M_ and Δ*y*_A,_ and the length change of half sarcomere as Δ*L*_hs_. Then, at the elastic equilibrium, they are given by

(24a) $$\Delta y_{\text{CB}}=-r_{\text{CB}}\Delta L_{\text{hs}}$$

(24b) $$\Delta y_{\text{M}}=-r_{\text{M}}\Delta L_{\text{hs}}$$

(24c) $$\Delta y_{\text{A}}-r_{\text{A}}\Delta L_{\text{hs}}$$

Naturally,

(25) $$r_{\text{CB}}+r_{\text{M}}+r_{\text{A}}=1$$

On the above assumption, only the length changes of the crossbridge and the myosin filament contribute to Phase T1. Then we have

(26) $$\Delta y_{\text{cT}1}=\Delta L_{\text{hs}}/C_{\text{CBT}}=\{r_{\text{CB}}/(r_{\text{CB}}+r_{\text{M}})\}\;\Delta L_{\text{hs}}$$

Thus,

(27) $$C_{\text{CBT}}=(r_{\text{CB}}+r_{\text{M}})/r_{\text{CB}}$$

As assumed above, the load change period is long enough and elastic changes of the crossbridge, myosin filament and actin filament contribute to Phase V1. Thus, Δ*y*_cV1_ is given by

(28) $$\Delta y_{\text{cV}1}=\Delta L_{\text{hs}}/C_{\text{CBV}}=\{r_{\text{CB}}/(r_{\text{CB}}+r_{\text{M}}+r_{\text{A}})\}\;\Delta L_{\text{hs}}=r_{\text{CB}}\Delta L_{\text{hs}}$$

Hence,

(29) $$C_{\text{CBV}}=1/{\text{r}}_{\text{CB}}$$

By using the values of *C*_CBT_ (Equation 22) and *C*_CBV_ (Equation 23), Equations 25, 27 and 29 give

(30a) $$r_{\text{CB}}=0.22$$

(30b) $$r_{\text{M}}=0.26$$

(30c) $$r_{\text{A}}=0.52$$

The extensibility ratios were investigated by X-ray diffraction by Huxley *et al.* [20] and Wakabayashi *et al.* [21]. The values reported by Wakabayashi *et al.* [21] are *r*_CB_ = 0.31, *r*_M_ = 0.27 and *r*_A_ = 0.42. Considering approximate nature of the theory and experimental errors, our values in Equation 30 are in reasonable agreement with the X-ray values.

In this section, it is assumed that elastic change of the actin filament does not take place during the fast length change in Phase T1. Then the elastic change of the actin filament should contribute to the next step, the tension recovery in Phase T2. This problem is discussed in next section.

## 5. Isometric Tension Transient

As noted in Section 4, Huxley [15] divided the transient responses to the sudden reduction of length into four phases. Time courses of these Phases are as follows. Phase 1 is instantaneous drop of tension. Phase 2 is rapid early tension recovery in next 1∼2 ms. Phase 3 is extreme reduction or even reversal of rate of tension recovery during next 5∼20 ms. Phase 4 is the gradual recovery of tension, with asymptotic approach to the isometric tension.

As in Section 4, we use the terms Phase Tn (n = 1, 2, 3, 4) in the tension transient to avoid confusion. Phases Tn are more related with molecular process rather than time sequence. Phase T1 is simultaneous drop of tension caused by elastic shortening of the crossbridge and myosin filament. Phase T2 is due to elastic shortening of the actin filament. Phase T3 is related with the U*_12_ transition, and divided into T3a and T3b. Phase T3a is the first part of Phase T3. Both Phases T2 and T3a contribute to the rapid early tension recovery (Phase 2 of Huxley). Phase T3b is the second part of T3 where the extreme reduction or reversal of rate of tension recovery occurs (Phase 3 of Huxley). Phase T4 is the gradual recovery of tension, with asymptotic approach to isometric tension (Phase 4 of Huxley). (There were misprints in Section 4.4 of the article [2], and the last three sentences of the section should be neglected.)

**Figure 11.**
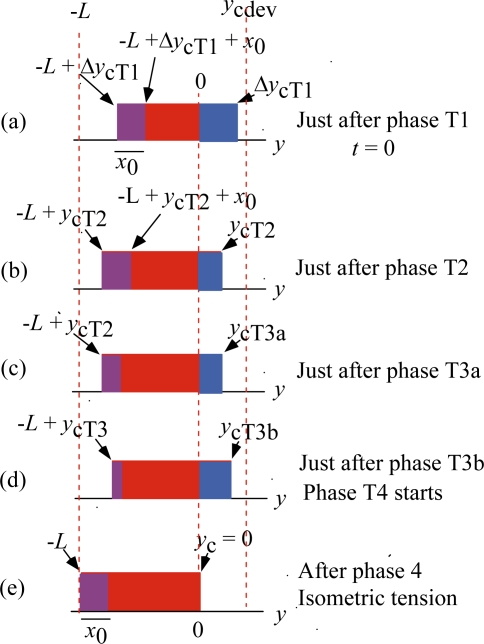
Changes of the *ρ* distribution during the isometric tension transient in the case of Δ*y*_cT1_ < *y*_cdev_. Magnitude of *y*_cdev_ is exaggerated for illustration.

**Figure 12.**
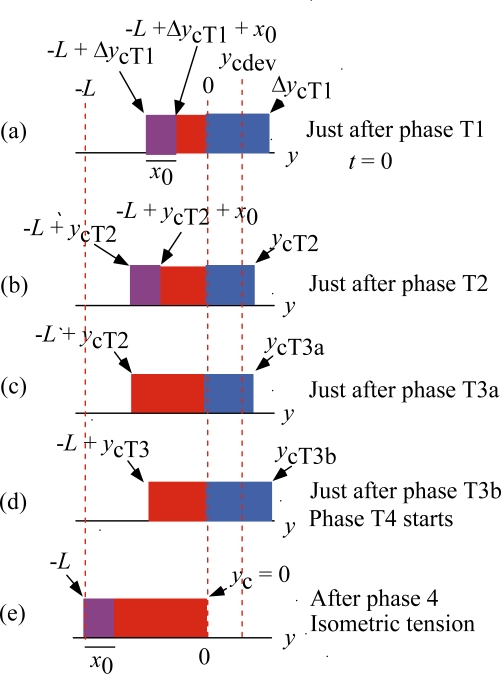
Changes of the *ρ* distribution during the isometric tension transient in the case of Δ*y*_cT1_ > *y*_cdev_.

Figures 11 and 12 illustrate how the rectangular *ρ* distribution changes during these Phases. Figure 11 is for the case of Δ*y*_cT1_ < *y*_cdev_ and Figure 12 is for the case of Δ*y*_cT1_ > *y*_cdev_, where Δ*y*_cT1_ is *y*_c_ just after Phase T1. (cf. Figure 10(b)).

As mentioned above, it is assumed that the elastic changes of the crossbridge and myosin filament occur in Phase T1 and then the elastic change of actin filament occurs in Phase T2. Figure 11(a) represents the states just after Phase T1. Figure 11(b) shows the state just after Phase T2. There is a time lag for the U*_12_ transition to occur, and its effect is neglected in Phases T1 and T2, so that the width of the violet area is kept the same as the isometric value *x*_0_.

As noted referring to Figure 5, the mean time (*τ*_step_) needed for the U* transition is relatively large (e.g., larger than 10 ms for *T*/*T*_0_ > 0.5). It is assumed that the potential barrier U*_12f_ (f: forward) is lower than U* as shown in Figure 3 (b) and thus the U*_12f_ transition starts before the U* transition. Phase T3a is regarded as the state that the U*_12f_ transition is present but the U* transition does not occur yet, while the U*_12f_ and U* transitions coexist in Phase T3b. In Phase 4, only the U* transition exists. Figure 11(c) and (d), respectively, show the states at the end of Phases T3a and T3b. The violet area in (c) is smaller than in (b) because a portion of the crossbridges in the violet area change their binding partners from A1 to A2 through U*_12f_ transition. Thus the red area increases and the stress is stored and the tension increases in Phase T3a. The U* transition starts at (c) and the stress stored during Phase T3a is released by the shift of the edge *y*_c_ from *y*_cT3a_ in (c) to *y*_cT3b_ in (d). The shift of the edge from *y*_cT3a_ (c) to *y*_cT3b_ (d) causes increase of the blue area and decrease of the violet area. These two effects tend to cancel each other and reduce change of the red area in the time course from (c) to (d), *i.e.*, in Phase T3b. The reduced change of the red area may cause the “extreme reduction of rate of tension recovery” mentioned at the beginning of this section. In Phase 4, the U* transition and the filament sliding continue and the state approaches to the tetanus state (e).

Figure 12 shows changes of the *ρ* distribution in the isometric tension transient for the case of Δ*y*_cT1_ > *y*_cdev_. Figure 12(a) and (b) are similar to Figure 11(a) and (b). Since Δ*y*_cT1_ > *y*_cdev_, the U*_12f_ transition does not leave the violet area and the red area significantly increases in (c). Then the U* transition starts and the *ρ* distribution shifts to the right as shown in (d). The red area significantly decreases from (c) to (d), and tension decreases and thus the “reversal of rate of tension recovery” cited at the beginning of this section is expected. The filament sliding continues in Phase 4, and the *ρ* distribution asymptotically approaches the isometric tetanus state (e).

**Figure 13.**
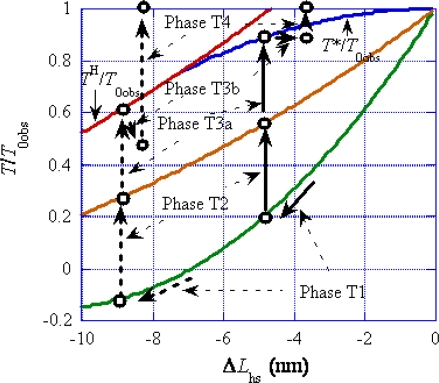
Phases in the isometric tension transient on the *T*/*T*_0obs_ *vs.* Δ*L*_hs_ relation. The thick black solid and dashed arrows, respectively, indicate Phases in the cases of Δ*y*_cT1_ < *y*_cdev_ and Δ*y*_cT1_ > *y*_cdev_. Actually the thick solid and dashed arrows are on the same vertical lines, respectively, except for the case of Phase T1.

Figure 13 illustrates changes in Phases T1∼T4 in another way by the *T*/*T*_0obs_ *vs.* Δ*L*_hs_ relation. The solid black arrows “Phase Tn” indicate an example of change of relative tension in Phase Tn for the case of Δ*y* < *y*_cdev_ and the dashed black arrows for the case of Δ*y* > *y*_cdev_. The edge Δ*y*_cT1_ in Figures 11(a) or 12(a) is resulted from the elastic changes of the crossbridge and myosin filament, and is given as a function of Δ*L*_hs_ by combining Equations 18 and 22:

(31) $$\Delta y_{\text{cT}1}=-\Delta L_{\text{hs}}/2.2$$

The tension variation *T*_T1_/*T*_0obs_ in Phase T1 can be calculated as a function of Δ*L*_hs_ by using Equations 20 and 31. Calculation results are shown by the green curve in Figure 13. The edge *y*_cT2_ in Figures 11(b) or 12(b) are the same quantity as Δ*y*_cV1_ in Figure 10(c), since they result from the elastic changes of the crossbridge, myosin filament and actin filament. Then, from Equations 19 and 23, we have

(32) $$y_{\text{cT}2}=-\Delta L_{\text{hs}}/4.6$$

Δ*y*_cT2_ = Δ*y*_cV1_ means *T*_T2_/*T*_0obs_ = *T*_V1_/*T*_0obs_. By using Equation 32 and replacing *T*_V1_ by *T*_T2_ and Δ*y*_cV1_ by Δ*y*_cT2_ in Equation 21,*T*_T2_/*T*_0obs_ can be calculated as a function of Δ*L*_hs_. The calculation result is given by the brown curve in Figure 13. In the steady filament sliding, there is the definite relation between the tension *T* and the parameter *y*_c_ as shown in Figure 8, where the red curve shows the relation for the Hill-type filament sliding and the blue curve shows the relation when the filament sliding deviates from the Hill-type. The parameter *y*_c_ in Figure 8 is related with Δ*y*_c_ in Figures 11 and 12 by the relation *y*_c_ = *y*_c0obs_ + Δ*y*_c_ since Δ*y*_c_ is the change of *y*_c_ from the value at the isometric tension (*y*_c0obs_). In the steady filament sliding, all elastic elongations of the crossbridge, the myosin filament and the actin filament contribute to the muscle elongation and thus Δ*y*_c_ (=*y*_c_ − *y*_c0obs_) is equal to −Δ*L*_hs_/4.6 as in Equation 32. The relative tension *T*/*T*_0_ in Figure 8 can be converted to *T*/*T*_0obs_ by using the ratio *T*_0obs_/*T*_0_ = 0.88 (Equation 2). Based upon these considerations, the red and blue curves in Figure 8 are reproduced with the same colors in Figure 13.

In Figure 13, the Phases are indicated by the solid black arrows “Phase Tn” in the case of Δ*y* < *y*_cdev_ (the case of Figure 11), and by the dashed black arrows in the case of Δ*y* > *y*_cdev_ (the case of Figure 12). The rapid tension change in Phase T1 occurs along the green curve as indicated by the solid or dashed arrows “Phase T1”. Phase T2 is a result of the elastic change of the actin filament and thus the arrows “Phase T2” start from the green curve and end on the brown curve. The main part of Phase T3a corresponds to the rapid tension increase from Figure 11(b) to (c) or from Figure 12(b) to (c). It is difficult to determine where Phase T3a turns into Phase T3b in Figure 13. Trial calculations, however, show that fairly good agreement can be obtained by assuming that Phase T3a ends at the blue curve or the red curve. Thus, in the case of Δ*y* < *y*_cdev_, the solid arrow “Phase T3a” is depicted with its tip on the blue curve. As discussed above, it is plausible that the red area in Figure 11(d) is nearly equal to that in (c). Taking this point into account, the solid arrow “Phase T3b” is depicted almost parallel to the abscissa, in accordance with the observation of the “extreme reduction of rate of tension recovery” noted at the beginning of this section. In the case of Δ*y* > *y*_cdev_, as discussed above, it is plausible that the red area significantly decreases from Figure 12(c) to (d). Accordingly, the dashed arrow “Phase T3b” is depicted downward, in accordance with the observation of the “reversal of rate of tension recovery ” noted at the beginning of this section. After Phase T3b, the tension *T* approaches *T*_0obs_ by the filament sliding as shown by the arrows “Phase T4”. The above argument on the solid and dashed arrows “Phase T3b” suggests that the reversal of rate of tension recovery occurs when |Δ*L*_hs_| is large. Supporting this conclusion, Figure 1 of the paper by Julian and Sollins [19] shows that the reversal of the rate occurs when |Δ*L*_hs_| is large.

**Figure 14.**
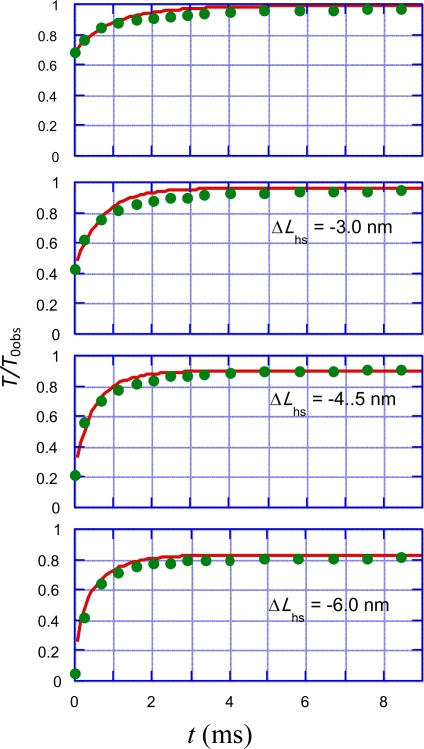
*T*/*T*_0obs_ as functions of time *t* for various length change steps Δ*L*_hs_ in the isometric tension transient. The origin of *t* is set as the moment that Phase T1 finishes. Green circles: Experimental data cited from Figure 23 of Ford *et al.* [16]. Red lines: Values of *T*_123a_/*T*_0obs_ calculated by using Equations 34, 35, 36 and the parameter values in Equation 38.

Ford *et al.* [16] reported experimental data on variation of *T*/*T*_0obs_ during 0–9 ms, as cited by green circles in Figure 14. The range of Δ*L*_hs_ was +1.5∼−6.0 nm in their experiment. Since our model is not applicable for positive Δ*L*_hs_, the case of +1.5 nm is omitted in Figure 14. Now we shall try to reproduce these results by calculation.

In Figure 14, values of *T*/*T*_0obs_ quickly increase within about 1 ms and then gradually approach to the value at 9 ms. The mean time duration needed for one U* transition is longer than 10 ms for *T*/*T*_0_ > 0.5 in Figure 5. Accordingly, effect of U* transition is not considered. The experimental length changes | Δ*L*_hs_ | are smaller than 7.0 nm as seen in Figure 14. Hence, the following discussion is done referring to the solid arrows which are in this | Δ*L*_hs_ | range. The tension changes in Phases T1, T2 and T3a are denoted as *T*_1_, *T*_2_ and *T*_3a_, respectively. Also symbols *T*_12_ and *T*_123a_ are defined as follows:

(33) $$T_{12}=T_{1}+T_{2}$$

(34) $$T_{123\text{a}}=T_{12}+T_{3\text{a}}$$

According to the scheme in Figure 13, the change of *T*_1_ is given by the green curve, and *T*_2_ changes from the green curve to the brown curve. In Figure 14, the origin of time *t* is set equal to 0 when the change of Phase T1 finishes. The parameters *C*_ini2_ and *C* _fin2_ are defined as the initial and final values of *T*_2_/*T*_0obs_, which are given by the green and brown curves, respectively in Figure 13. If *T*_2_ is approximately expressed by a single decay constant *τ*_T2_, *T*_12_/*T*_0obs_ is given by,

(35) $$T_{12}/T_{0\text{obs}}=C_{\text{fin}2}-(C_{\text{fin}2}-C_{\text{ini}2})\text{exp}(-t/\tau_{\text{T}2})$$

The constant *τ*_T2_ is related with the elastic change of the actin filament and is independent of Δ*L*_hs_. The solid arrow “Phase T3b” is drawn between the brown curve and the blue curve in Figure 13, so that the magnitude of tension of *T*/*T*_0obs_ in T3b is given by the difference between these curves. It is uncertain when the U*_12_ transition and thus Phase T3a start. To make calculation simple, it is assumed that it starts at the same moment as Phase T2, *i.e.*, at *t* = 0. Also the change of *T*_3a_ is approximately expressed with a single decay constant *τ*_T3a_:

(36) $$T_{3\text{a}}/T_{0\text{obs}}=(C_{\text{fin}3\text{a}}-C_{\text{ini}3\text{a}})\{1-\text{exp}(-t/\tau_{\text{T}3\text{a}})\}$$

where (*C*_ini3a_ − *C*_fin3a_) represents the magnitude of the tension variation. *C*_ini3a_ and *C*_fin3a_ are given by the brown and blue curves, respectively in Figure 13.

Now the problem is how the parameter *τ*_T3a_ changes with Δ*L*_hs_. In the discussion on the force-velocity relation in [2], the average time *t*_c_ for a myosin head at *y*_c_ to cross over the potential barrier U*(*y*_c_) is expressed as *t*_c_ = (1/*A*) exp (U*(*y*_c_)/*kT*) (Equations 4–2–1 in [2]), and U*(*y*_c_) is expressed by U*(*y*_c_) = U*_0_ − *by*_c_ (Equations 4–2–2 in [2]). These formulae give *t*_c_ = *B* exp (−*cy*_c_) where *B* and *c* = *b*/*kT* are constants. In Figure 11(b) the boundary between the violet and red areas is indicated as −*L* + *y*_cT2_ + *x*_0_. The relative relation between this boundary and A1 is similar to that between *y*_c_ and A2. Then, in analogy to the above relation, with constants *B*’ and *c*’, the relation _T3a_ = *B*’ exp (−*cy*_c_’) is expected as an approximate expression, where *y*_c_’ = −*L* + *y*_cT2_ + *x*_0_ (Figure 11(b)). Then, as *L* and *x*_0_ are constants, *τ*_T3a_ in Equation 36 is approximately given by *τ*_T3a_ = *B*exp(−*c’y*_cT2_), where *c*’ is constant. Since *y*_cT2_ = −Δ*L*_hs_/4.6 (Equation 32), this relation can be rewritten as,

(37) $$\tau_{\text{T}3\text{a}}=A_{3\text{a}}\;\text{exp}(c_{3\text{a}}\Delta L_{\text{hs}})$$

where *A*_3a_ and *c*_3a_ are constants. This relation implies that *τ*_T3a_ becomes small and the tension variation becomes fast when | Δ*L*_hs_ | increases.

Trial calculations were done for *T*_123a_ = *T*_12_ + *T*_3a_ (Equation 34) to explain the experimental observations by changing parameters *τ*_T2_, *A*_3a_ and *c*_3a_ in Equations 35, 36 and 37. Fairly good agreement with the experimental data is obtained as shown in Figure 14 by using the following parameter values:

(38a) $$\tau_{\text{T}2}=0.7\;\text{ms}$$

(38b) $$A_{3\text{a}}=3\;\text{ms}$$

(38c) $$c_{3\text{a}}=0.5\;(1/\text{nm})$$

In Section 4, it is assumed that the elastic change of the actin filament occurs 0.4–1 ms after the length changes of the crossbridge and myosin filament. The time constant *τ*_T2_ = 0.7 ms is in consistency with this assumption.

## 6. Isotonic Velocity Transient

Isotonic velocity transients were studied by Civan and Podolsky [17], Huxley *et al.* [15] and Sugi and Tsuchiya [22,23]. A muscle was stimulated and initially held at a constant length. It was then released suddenly and allowed to shorten under a constant load. In this section, discussion will refer to the experimental data presented in Figure 3 of the article by Civan and Podolsky [17]. Responses of muscle to the sudden load change are classified into four Phases by Huxley [15]. Analogously, we use terminology “Phase Vn” as mentioned in Section 4. While Phase number of Huxley is related with time sequence of the length changes, Phase Vn is more related with molecular processes rather than the time sequence. Phase V1 is the length change which simultaneously occurs with load change. Phase V2 is rapid early shortening. Phase V3 is extreme reduction or reversal of shortening speed. Phase V4 is responsible to the fact that the filament sliding velocity temporarily becomes larger than the steady value. Phases V2, V3 and V4 overlap each other in their time courses.

**Figure 15.**
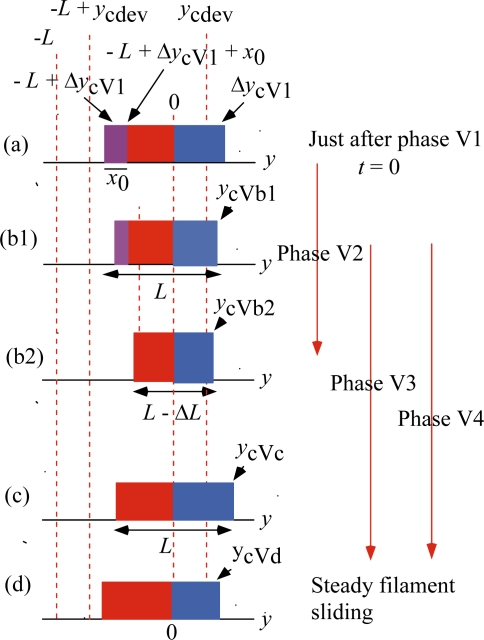
Changes of the *ρ* distribution in the isotonic velocity transient when Δ*y*_cV1_ > y_cdev_.

Figure 15 shows changes of the *ρ* distribution in these four Phases. Figure 15 (a) is the *ρ* distribution just after Phase V1. Since *y*_c_ = 0 at the isometric tension (cf. Figure 10 (a)), *y*_c_ in Figure 15 (a) is equal to the variation from the isometric tension, Δ*y*_cV1_ (cf. Figure 10 (c)). We shall discuss the case of Δ*y*_cV1_ > *y*_cdev_, since most experiments on the velocity transient were done for Δ*y*_c_ > *y*_cdev_.

In Figure 15, the vertical red arrows show time courses of Phases V2, V3 and V4. They overlap each other indicating the overlap of their time courses. The width of the violet area *x*_0_ in Figure 15(a) is the same as in the isometric tension since the U*_12_ transition does not occur yet. Then the U*_12_ transition starts, *i.e.*, Phase V2 starts. As mentioned above, there is a time delay in occurrence of the U* transition and thus of the filament sliding. Figure 15 (b1) shows the state in midway of Phase V2, where the U* transition starts. In (b1), a portion of the violet area has turned into red due to the U*_12f_ transition and internal stress increases, which causes shrinkage of the *ρ* distribution, and thus of the muscle. At (b2), the U*_12f_ transition is over and all the violet area is turned into the red area as Δ*y*_cV1_ > *y*_cdev_. The process from (a) to (b2) is related with the U*_12_ transition and is called Phase V2, where the red area increases and the internal stress increases. The width of *ρ* distribution changes from *L* in (a) to *L*-Δ*L* in (b2). Accordingly, a fast shortening of the muscle is expected.

During Phase V2 the U* transition starts at (b1). There is a mutual interaction between the U* transition and the internal stress. The U* transition causes the filament sliding, releases the internal stress and tends to make *y*_c_ larger. The internal stress tends to expand the *ρ* distribution, increases *y*_c_ and accelerates the U* transition. The former process is called Phase V3. The latter process is called Phase V4. The mutual interaction almost disappears leaving a large value of *y*_c_ at (c). Then the frequency of the U* transition and *y*_c_ change toward their steady values from (c) to (d), where the *ρ* distribution at the steady filament sliding is shown. Phases V3 and V4 occur in parallel starting with the U* transition at (b1) and end near (d).

**Figure 16.**
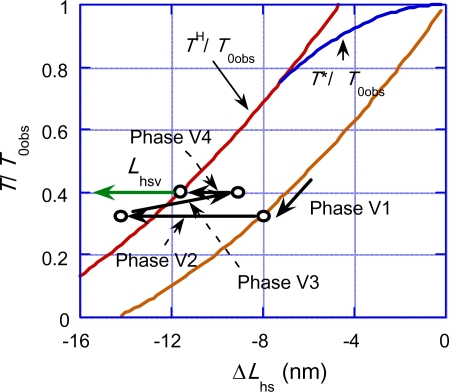
Phases in the isometric tension transient on the *T*/*T*_0obs_ *vs.* Δ*L*_hs_ relation. The black arrows indicate an example of changes of the relevant Phases in the case of Δ*y*_cV1_ > *y*_cdev_. Actually the arrows are on the same horizontal lines, except for the case of Phase V1.

Figure 16 illustrates changes in Phase V1∼V4 in another way. This figure is drawn considering the case of small *T*/*T*_0obs_. (An example of the experimental data for small *T*/*T*_0obs_ can be seen in Figure 18 (c) given later where Δ*T*/*T*_0obs_ = 0.87, *i.e.*, *T*/*T*_0obs_ = 0.13.) The red, blue and brown curves are the same as the curves of the same colors in Figure 13. The black arrows indicate an example of changes of Δ*L*_hs_ in the four Phases. As discussed above, there is the overlap between the Phases, and the arrow “Phase Vn” indicates the Phase which mainly contributes to the process.

The length change in Phase V1 occurs along the brown curve, since the elastic changes of the crossbridge, the myosin filament and the actin filament contribute to this change. The arrow “Phase V2” corresponds to the rapid decrease of muscle length from Figure 15 (a) to (b2). The length of the arrow “Phase V2” is tentatively depicted as 6 nm (the same order of magnitude of the rapid drop of 5 nm in Figure 18 (c) given later). As discussed above, the time courses of Phases 3 and 4 overlap each other. The direction of arrow “Phase V3” is reversed, representing the muscle elongation from Figure 15 (b2) to (c). Through “Phase 4”, the system approaches to the red curve corresponding to the change from (c) to (d) in Figure 15. The green arrow “*L*_hsv_” symbolically represents the steady filament sliding corresponding to the state in Figure 15 (d).

Now let us numerically reproduce the experimental data presented in Figure 3 of the paper by Civan and Podolsky [17]. The length change per half sarcomere at the steady filament sliding is denoted as *L*_hsv_ which corresponds to the dashed line in Figure 3 of [17]. Discussion will be done referring to *L*_hsv_ expressed by

(39) $$L_{\text{hsv}}=-vt$$

Here *v* is the sliding velocity determined by the experimental data in [17].

The length change caused by the U*_12_ transition in Phase V2 is denoted as *L*_hs2_. The speed of *L*_hs2_ depends upon the frequency of U*_12_ transition and will be large at the beginning and gradually decay. Its time course is approximately expressed with decay constant *τ*_V2_ by

(40) $$L_{\text{hs}2}=-B_{\text{V}2}(1-\text{exp}(-t/\tau_{\text{V}2}))$$

where *B*_V2_ is a constant.

As discussed above, there is the mutual interaction between the U* transition and the internal stress. (a) The U* transition causes the filament sliding which releases the internal stress. (b) The internal stress tends to expand the *ρ* distribution, pushes *y*_c_ forward and accelerates the U* transition. The interaction (a) is discussed first. The interaction (b) will be discussed later in relation with Phase 4.

The interaction (a) releases the internal stress and thus elongates the muscle. As shown in Figure 15, there is an overlap between Phase V2 and V3, and thus there is an overlap between length change *L*_hs2_ and the length change due to the interaction (a). The combined length change is denoted as *L*_hs23_, which is expressed by multiplying *L*_hs2_ by exp(−*t*/*τ*_V3_):

(41) $$L_{\text{hs}23}=-B_{\text{V}2}\{1-\text{exp}(-t/\tau_{\text{V}2})\}\;\text{exp}(-t/\tau_{\text{V}3})$$

**Figure 17.**
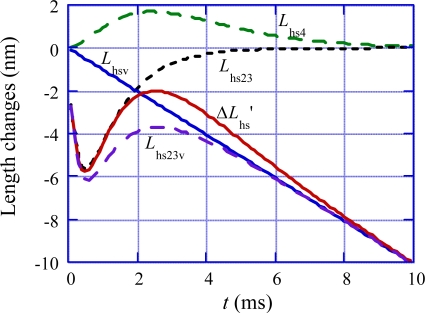
Characteristics of *L*_hs4_, *L*_hsv_, *L*_hs23_, *L*_hs23v_ and Δ*L*_hs_’ are illustrated in the case of Δ*T*/*T*_0obs_ = (*T*_0obs_ − *T*)/*T*_0obs_ = 0.87, by using the equations and parameter values given in text.

An example of *L*_hs23_ is shown by the dotted black curve in Figure 17 to demonstrate its characteristics, together with *L*_hsv_ (the blue line).

The decay times *τ*_V2_ and *τ*_V3_ should be functions of *T*/*T*_0obs_ or *v*. The magnitude of *τ*_V2_ is proportional to the time duration of occurrence of the U*_12_ transition. Analogously to the manner to derive Equation 37, the relation *τ*_T3a_ = *B*exp (−*cy*_c_’) is expected as an approximate expression, where *y*_c_’ is the *y* at the boundary between the violet area and red area in Figure 15(a), where *y*_c_’ = −L + Δ*y*_cV1_+*x*_0_. The tension *T* becomes smaller as Δ *y*_cV1_ becomes larger. As a simple approximation, Δ*T* = *T*_0obs_ − *T* is set proportional to Δ *y*_cV1_. Then the decay time *τ*_V2_ is given approximately by

(42) $$\tau_{\text{V}2}=a_{\text{V}2}\;\text{exp}(c_{\text{V}2}T/T_{0\text{obs}})$$

where *a*_V2_ and *c*_V2_ are constants. The time constant *τ*_V3_ in Phase V3 is related with the U* transition, and its magnitude seems to be an order of *τ*_step_ = *L*/*v* (Equation 5). Hence *τ*_V3_ is set as

(43) $$\tau_{\text{V}3}=b_{\text{V}3}(L/v)$$

where *b*_V3_ is a constant.

To see the characteristics of combination of *L*_hsv_ and *L*_hs23_, *L*_hs23v_ is defined by

(44) $$L_{\text{hs}23\text{v}}=L_{\text{hsv}}+L_{\text{hs}23}$$

An example of *L*_hs23v_ is illustrated by the dashed violet curve in Figure 17.

Now let us consider about the part (b) of the interaction that the internal stress tends to expand the *ρ* distribution, pushes *y*_c_ forward and accelerates the U* transition. This effect causes muscle elongation. In Figure 15, this effect is illustrated by depicting *y*_cVc_ in (c) larger than *y*_cVd_ in (d). In Phase 4, *y*_cVc_ changes into *y*_cVd_ and the filament sliding approaches to the steady value. As shown by the overlap of the arrows of Phases V3 and V4 in Figure 15, these effects overlap with each other. The length change in Phase V4 is denoted as *L*_hs4_. In analogy to the expression of *L*_hs23_ (Equation (41)), *L*_hs4_ is approximately expressed by

(45) $$L_{\text{hs}4}=B_{\text{V}4}{\left\{1-\text{exp}(-t/\tau_{\text{V}4})\right\}}^{2}\;\text{exp}(-t/\tau_{\text{V}5})$$

The characteristics of *L*_hs4_ expressed by this equation are illustrated by the dashed green curve in Figure 17. The internal stress is zero at *t* = 0 and increases by the term {1 − exp(−*t*/*τ*_V4_)}^2^. The term exp(−*t*/*τ*_V5_) corresponds to the decrease of the muscle length due to the filament sliding. Concerning the magnitude of *B*_V4_, it is plausible that the internal stress pushes *y*_c_ forward more effectively when the internal stress rapidly increases, *i.e.*, when the frequency of the U*_12_ transition rapidly increases. The frequency of the U*_12_ transition is given by the reciprocal of the duration *τ*_V2_ given by Equation 42. Thus *B*_v4_ is expressed by

(46) $$B_{\text{V}4}=G_{\text{V}4}/\tau_{\text{V}2}$$

where *G*_v4_ is a constant. The time constants *τ*_V4_ and *τ*_V5_ will be mainly related with the U* transitions, and in analogy to Equation 43 they are expressed as

(47) $$\tau_{\text{V}4}={\text{b}}_{\text{V}4}(L/v)$$

(48) $$\tau_{\text{V}5}={\text{b}}_{\text{V}5}(L/v)$$

where *b*_4_ and *b*_5_ are constant.

Since the sum of *L*_hsv_, *L*_hs23_ and *L*_hs4_ is the total length change, it is denoted as Δ*L*_hs_’

(49) $$\Delta L_{\text{hs}}’=L_{\text{hs}23}+L_{\text{hs}4}+L_{\text{hsv}}$$

Note that Δ*L*_hs_’ is the length change from the moment when Phase 1 finishes, while the abscissa Δ*L*_hs_ in Figure 16 includes the length change in Phase 1.

**Figure 18.**
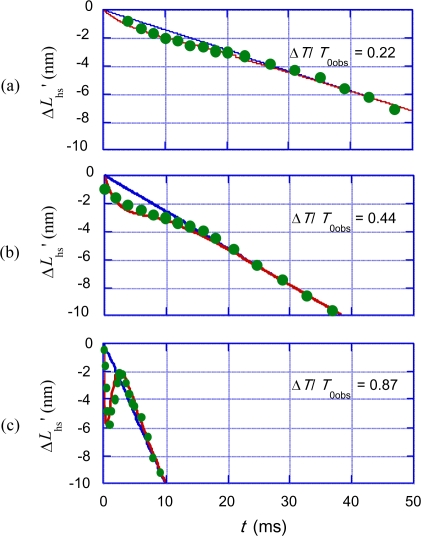
Length change Δ*L*_hs_’ *vs.* time *t* for three values of Δ*T*/*T*_0obs_= (*T*_0obs_ − *T*)/*T*_0obs_. **(a)** Δ*T*/*T*_0obs_ = 0.22; **(b)** Δ*T*/*T*_0obs_ = 0.44; **(c)** Δ*T*/*T*_0obs_ = 0.87. Green data points are obtained from Figure 3 of the paper by Civan and Podolsky [17]. Blue straight lines are *L*_hsv_ = −*vt* (Equation 39). Red curves are Δ*L*_hs_’ calculated by using Equation 49 and the parameter values in Equation 50.

Time course of Δ*L*_hs_’ was calculated with various trial values of the parameters looking for good agreement with the experimental data in the cases of (*T*_0obs_ − *T*)/*T*_0obs_ = 0.22, 0.44 and 0.87. Calculation results with the following parameter values are shown in Figure 18.

(50a) $$B_{\text{V}2}=12\;\text{nm}$$

(50b) $$a_{\text{V}2}=0.14\;\text{ms}$$

(50c) $$c_{\text{V}2}=7.6$$

(50d) $$G_{\text{V}4}=15.0\;\text{pm}/\text{s}$$

(50e) $$b_{\text{V}3}=0.20$$

(50f) $$b_{\text{V}4}=0.8$$

(50g) $$b_{\text{V}5}=0.3$$

Characteristic features of the series of experimental data are fairly well reproduced by the calculation. The drastic change of the curve shape for different Δ*T*/*T*_0obs_ is mainly due to the *T* dependence of the frequency of the occurrence of the U*_12_ transition, *i.e.*, the relation *τ*_V2_ = *a*_V2_exp(*c*_V2_*T*/*T*_0obs_) (Equation 42).

## 7. Deviation from Hill-Type Force-Velocity Relation

Let us consider the deviation from Hill-type force-velocity relation in connection of the U*_12_ transition. The deviation is determined by change of crossbridge population in well A2 and the U* potential barrier height. The maximum ratio of crossbridge population in A1 per that in A2 is given by *x*_0_/(*L*−*x*_0_) in Figure 7 (b3). This ratio is not very large and the effect of population change is neglected in the following approximate formulation.

The relative tensions *T*^H^/*T*_0_ and T*/*T*_0_ are given as functions of *y*_c_^H^ and *y*_c_* in Figure 8. The same relationship can be expressed by giving *y*_c_^H^ and *y*_c_* as functions of *T*/*T*_0_. From Equation 8, we have

(51) $$y_{\text{c}}^{\text{H}}=L[\kappa_{\text{f}}-\{\kappa_{\text{f}}^{2}-(\kappa_{\text{f}}-\kappa_{\text{b}}){\left\{\kappa_{\text{f}}-2p_{0}T^{\text{H}}/(T_{0}L)\right\}}^{1/2}\}]/(\kappa_{\text{f}}-\kappa_{\text{b}})$$

From Equation 12, we have

(52) $$y_{c}^{*}={\left[\{b-(T^{*}/T_{0})\}/a\right]}^{1/2}$$

**Figure 19.**
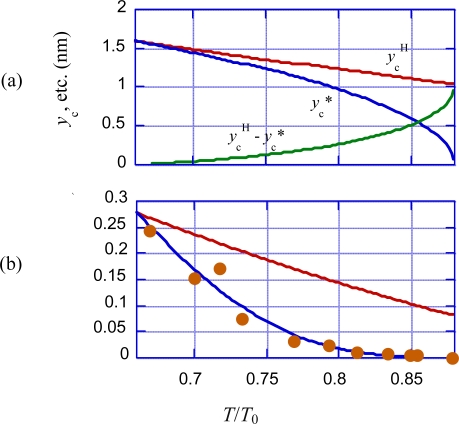
Explanation of the deviation of the force-velocity relation from Hill-type. **(a)** *y*_c_^H^, *y*_c_*, *y*_c_^H^ − *y*_c_* as functions of *T*/*T*_0_, calculated by Equations 51 and 52; **(b)** Brown circles: Experimental data of the sliding velocity per half sarcomere cited from Fig, 3A of the paper by Edman [12]. Red curve: *v*^H^, the Hill-type velocity calculated in [2]. Blue curve: v*, the velocity in the presence of the U*_12_ transition calculated by Equations 53 and 54.

These relations and *y*_c_^H^ − *y*_c_* are shown as functions of *T*/*T*_0_ = *T*^H^/*T*_0_ = T*/*T*_0_ in the range between *T*_dev_/*T*_0_ and *T*_0obs_/*T*_0_ in Figure 19 (a).

The filament sliding velocity of Hill-type is denoted as *v*^H^ and the velocity in the presence of the U*_12_ transition as v*. The period for the crossbridge to move over *L* is given as *τ*_step_ = *L*/*v*^H^ in Figure 5, which should have a similar nature as the time duration of U* transition, *t*_c_ = (1/*A*)exp(U*/*kT*) (Equation 4–2–1 in [2]), where *A* is a constant and U* is the potential barrier for the U* transition. Thus *v*^H^ = *L*/*τ*_step_ is approximately proportional to 1/exp(U*/*kT*). Here U* is the potential barrier height when there is no U*_12_ transition. The difference *y*_c_^H^ − *y*_c_* shown in Figure 19 (a) can be regarded as a measure of the magnitude of structural change in MA_3_. The potential barrier U* should be affected by this structural change, and the change of the U* height is approximately set as b_V_(*y*_c_^H^ − *y*_c_*) where *b*_V_ is a constant proportional to 1/*kT*. Then exp(U*/*kT*) changes into exp((U*/*kT*) + (*b*_V_(*y*_c_^H^ − *y*_c_*)) in *T*_dev_ < *T* < *T*_0obs_. Then, if we put *v*^H^ = *C*/exp(U*/*kT*) and v* = *C*/exp((U*/*kT*) + *b*_v_(*y*_c_^H^ − *y*_c_*)) with a constant *C*, we have

(53) $$v^{*}=v^{\text{H}}/\text{exp}(b_{\text{V}}(y_{\text{c}}^{\text{H}}-y_{\text{c}}^{*}))$$

Since *y*_c_^H^ = *y*_c_* = *y*_cdev_ (cf. Figure 8), Equation 53 gives v* = *v*^H^ at *y*_cdev_, as required. The blue curve in Figure 19(b) shows results of calculation by Equation 53 with the parameter value

(54) $$b_{\text{V}}=7.9\;(1/\text{nm})$$

Agreement with the experimental data is fairly good. Accordingly, the deviation from Hill-type force-velocity relation is mainly due to the change of the potential barrier height U* caused by occurrence of U*_12_ transition.

## 8. Summary and Discussion

In our previous paper [2] (the first part of this Remarks series), difficulty of the power stroke model is pointed out and a new model is proposed to avoid the difficulty. In the model, it is proposed that about 41% of the myosin heads are bound to actin filament and each bound head forms complex MA_3_ with three actin molecules. The complex MA_3_ translates along the actin filament changing its partner actin molecules in cooperation with U* transition. This model well explains the properties in the steady filament sliding such as the tension-dependence of the muscle stiffness, the Hill-type force velocity relation and the tension-dependence of energy liberation rate. In the present paper, the isometric tension transient and isometric velocity transient are studied based upon the model. Statistical ensemble of crossbridges is considered and the binding probability density *ρ* is introduced. On rectangular *ρ* approximation, the edge of the rectangle, *y*_c_ determines dynamic properties of muscle in cooperation with U* and U*_12_ transitions. The internal structure of the MA_3_ complex becomes temporally unstable by the sudden length change or by the sudden load change. The complex muscle behaviors observed in these transients are related with the process that the disturbed internal structure returns to its stationary state.

Results reported in the present paper are summarized as follows.

1. The tension variations in the first Phases in the isometric tension transient (Ford *et al.* [16]) and the isotonic velocity transient (Civan and Podolsky [17]) are well explained as shown in Figure 9.
2. Ratios of extensibilities of crossbridge, myosin filament and actin filament are estimated as 0.22, 0.26 and 0.52 (Equation 30), in reasonable agreement with the approximate values (0.31, 0.27, 0.42) determined by X-ray diffraction by Wakabayashi *et al.* [21].
3. The experimental data on the isometric tension transient reported by Ford *et al.* [16] are fairly well explained as shown in Figure 14.
4. The characteristic features of muscle in the isotonic velocity transient observed by Civan and Podolsky [8] are fairly well explained as shown in Figure 18.
5. The deviation from the Hill-type force-velocity relation observed by Edman [12] is reproduced as shown in Figure 19(b).

It should be noted that the above-mentioned agreements between experimental data and calculation results are obtained by using the muscle stiffnesses *κ*_f_ and *κ*_b_ determined in [2], whose numerical values are given in Appendix.

The obtained results suggest that the ideas of the ensemble of crossbridges and the rectangular *ρ* approximation are useful tools in theoretical studies of muscle contraction.

In Figure 8, tangent of the T*/*T*_0_ *vs. y*_c_* relation (the blue curve) is 0 at *y*_c_* = 0. Accordingly, small fluctuation of tension around *T*_0obs_ can produce significant variation of the muscle length. In this connection, the spontaneous oscillatory contraction (SPOC) of muscle (cf. the review by Ishiwata and Yasuda [24]) seems interesting. As mentioned in [24], it is a possibility that SPOC has some relation with the activities of cardiac muscles (cf. [25]).

## Appendix Symbols and Their Values

*kT*: 3.77 × 10^−21^ at 0 °C.
*κ*_f_: stiffness of an elongated crossbridge. 2.80 pN/nm (Equation 4–1–13 in [2]).
*κ*_b_: stiffness of a shrinked crossbridge. 0.26 pN/nm (Equation 4–1–14 in [2]).
*L*: period of binding sites of the myosin head along the actin filament, 5.46 nm.
*L*_hs_: length of the half sarcomere.
*p*: mean force produced by one crossbridge.
*p*_0_: maximum *p* in the extended Hill-type force-velocity relation.
*p*_0obs_: *p* in isometric contraction. 5.7 pN (Equation 2–2–3 in [2]).
*r*: ratio = (number of myosin heads attached to actin filaments)/(total number of myosin heads) in contracting muscle. 0.41 (Equation 3–1–1 in [2]).
*r*_CB_: extensibility ratio of crossbridge. 0.22 (Equation 30a).
*r*_M_: extensibility ratio of myosin filament. 026 (Equation 30b).
*r*_A_: extensibility ratio of actin filament. 0.52 (Equation 30c).
*s*: sarcomere length of muscle with a full filament overlap. 2.10 μm [3].
*T*: tension (*P* is used for *T* in [2]).
*T*_0_: maximum *T* in the extended Hill-type force-velocity relation (*P*_0_ in [2]).
*T*_0obs_: isometric tension of muscle with a full filament overlap. 4.1 × 10_5_ N/m_2_ [3]. *T*_0obs_/*T*_0_ = 0.88 (cf. Figure 1).
*T*_dev_: *T* at which the velocity starts to deviate from Hill-type force-velocity relation.
*T*_dev_/*T*_0_ = 0.66 (cf. Figure 1).
*T**: observed tension for *T* > *T*_dev_.
*v*_max_: velocity of the filament sliding under no load on muscle. 2.36 μm/s at 1.8 °C [3].
*y*: srinkage shortening of the crossbridge (cf. Figure 4).
*y*_c_: maximum *y* in the rectangular *ρ* distribution.
*y*_c_(0): *y*_c_ for *v* = *v*_max_. 4.2 nm (Equation 4–1–11 in [2]).
*y*_c0_: *y*_c_ for *v* = 0 in the Hill-type force-velocity relation. 0.73 nm (Equation 4–1–12 in [2]).
*y*_cdev_: *y*_c_ for *T*_dev_/*T*_0_ = 0.66. 1.60 nm (Figure 8).
*y*_c0obs_: *y*_c_ for *T*_0obs_/*T*_0_ = 0.88. 1.03 nm (Figure 8).
*y*_c_*: *y*_c_ observed in the presence of the *U**_12_ transition (Figure 7).

## References

[1] Mitsui T (1999). Induced potential model of muscular contraction mechanism and myosin molecular structure. Adv. Biophys.

[2] Mitsui T, Ohshima H (2008). Remarks on muscle contraction mechanism. Int. J. Mol. Sci.

[3] Oshima K, Takezawa Y, Sugimoto Y, Kobayashi T, Irving TC, Wakabayashi K (2007). Axial dispositions and conformations of myosin crossbridges along thick filaments in relaxed and contracting states of vertebrate striated muscles by X-ray fiber diffraction. J. Mol. Biol.

[4] Knupp C, Offer G, Ranatunga KW, Squire JM (2009). Proving muscle myosin motor action: X-ray (M3 and M6) interference measurements report motor domain not lever arm movement. J. Mol. Biol.

[5] Andreeva AL, Andreev OA, Borejdo J (1993). Structure of the 265-kilodalton complex formed upon EDC cross-linking of subfragment 1 to F-actin. Biochemistry.

[6] Podolsky RJ, Onge SS, Yu L (1976). X-ray diffraction of actively shortening muscle. Proc. Natl. Acad. Sci. USA.

[7] Huxley HE, Sugi H, Pollack GH (1979). Time resolved X-ray diffraction studies in muscle. Cross-bridge Mechanism in Muscle Contraction.

[8] Huxley HE, Kress M (1985). Crossbridge behaviour during muscle contraction. J. Musc. Res. Cell Motility.

[9] Yagi N, Takemori S, Watanabe M (1993). An X-ray diffraction study of frog skeletal muscle during shortening near the maximum velocity. J. Mol. Biol.

[10] Hill AV (1938). The heat of shortening and the dynamic constants of muscle. Proc. Roy. Soc.

[11] Hill AV (1964). The effect of load on the heat of shortening muscles. Proc. Roy. Soc. B.

[12] Edman KAP (1988). Double-hyperbolic force-velocity relation in frog muscle fibres. J. Physiol.

[13] Eyring H, Atkins PW (1998). The Eyring equations. Physical Chemistry.

[14] Huxley AF, Simmons RM (1971). Proposed mechanism of force generation in striated muscle. Nature.

[15] Huxley AF (1974). Muscular contraction. J. Physiol.

[16] Ford LE, Huxley AF, Simmons RM (1977). Tension responses to sudden length change in stimulated frog muscle fibres near slack length. J. Physiol.

[17] Civan MM, Podolsky RJ (1966). Contraction kinetics of striated muscle fibres following quick changes in load. J. Physiol.

[18] Irving M (1955). Give in the filament. Nature.

[19] Julian FJ, Sollins MR (1975). Variation of muscle stiffness with force at increasing speeds of shortening. J. Gen. Pysiol.

[20] Huxley HE, Stewart A, Sosa H, Irving T (1994). X-ray diffraction measurements of the extensibility of actin and myosin ffilaments in contracting muscle. Biophys. J.

[21] Wakabayashi K, Sugimoto Y, Tanaka H, Ueno Y, Takazawa Y, Amemiya Y (1994). X-ray diffraction evidence for the extensibility of actin and myosin filaments during muscle contraction. Biophys. J.

[22] Sugi H, Tsuchiya T (1981). Isotonic velocity transients in frog muscle fibres following quick changes in load. J. Physiol.

[23] Sugi H, Tsuchiya T (1981). Enhancement of mechanical performance in frog muscle fibres after quick increases in load. J. Physiol.

[24] Ishiwata S, Yasuda K (1993). Mechano-chemical coupling in spontaneous oscillatory contraction of muscle. Phase Trans.

[25] Bers D (2001). Exitation contraction coupling and cardiac contractile force. Development in Cardiovascular Medicine.

